# Cystic tuberculosis of the scapula in a young boy: a case report and review of the literature

**DOI:** 10.4076/1752-1947-3-7412

**Published:** 2009-08-05

**Authors:** Deepali Jain, Vijay K Jain, Yashwant Singh, Satish Kumar, Deepak Mittal

**Affiliations:** 1 Department of Pathology, Maulana Azad Medical College, New Delhi, India; 2 Departments of Orthopaedics & Radiodiagnosis, Dr. Ram Manohar Lohia Hospital, New Delhi 110001, India

## Abstract

**Introduction:**

Tuberculosis of the flat bones is rare and only a small percentage involves the scapular bone.

**Case presentation:**

We report a rare case of tuberculosis of the scapula in a 14-year-old. Diagnostic clues include lytic areas with low density seen in the body of the scapula involving a glenoid margin associated with typical clinical features. Treatment should include a regimen of four antitubercular drugs along with surgical debridement if required.

**Conclusion:**

Although rare, tuberculosis should be suspected in patients presenting with a chronic sinus in the scapular region, particularly in the developing world.

## Introduction

Tuberculosis (TB) has been a health concern for several thousand years. Only a small number of patients with tuberculosis will have osteoarticular involvement [1]. Less than one percent of all osteoarticular TB affects the shoulder, a fraction of it involving the scapular bone itself [2]. To the best of our knowledge, only eleven cases of scapular tuberculosis have been reported to date [3]-[12]. We present the 12th case, occurring in a pediatric patient, which has been described only twice before in the English literature ([Table 1]) [5,10].

**Table 1:** Review of the literature of previously reported cases of TB of the scapula

S.N	Author year	No. of patients	Age/sex	Location	Side	Presenting complaints	Other sites	Treatment
1	Lafond 1958 [3]	One	NA	NA	NA	NA	NA	NA
2	Martini et al. 1986 [4]	One	NA	Acromian	NA	NA	NA	NA
3	Shannon et al. 1990 [5]	One	4/male	Scapula	Lt	Pain and swelling of the left shoulder	Isolated with Rt ileum involvement, multifocal cystic	ATD
4	Mohan et al. 1991 [6]	One	23/female	Body of scapula	Rt	Pain and swelling	Isolated	Drainage and ATD
5	Gusati et al. 1997 [7]	One	NA	Spine of scapula	NA	Pain	Isolated	Surgery and ATD
6	Vohra et al. 1997 [8]	One	NA	Body of scapula	NA	NA	Isolated	NA
7	Kam et al. 2000 [9]	Two	31/male 22/female	Acromian, Lareral border of scapula	Rt Rt	1) Pain and swelling 2) Incidental finding	Isolated, Multifocal (T12 and L2 vertebrae; upper part of the right sacroiliac Joint)	Debridement and curettage and ATD, ATD alone
8	Greenhow and Weintrub 2004 [10]	One	14/female	Inferior aspect of the left scapula	Lt	Enlarging, nontender mass	Cystic lesion with a soft tissue component, located dorsal to the left scapula	Scapular mass excision
9	Stones and Schoeman 2004 [11]	One	42/male	Scapula	NA	Discharging sinus	As apart of multimodal tuberculosis involving maxilla, parital bones and spine	Died
10	Husen et al. 2006 [12]	One	18/male	Spine of scapula near neck	Lt	Diffuse pain	Isolated	ATD
11	Present case 2007	One	14/male	Body of scapula involving glenoid margin	Rt	Pain swelling and discharging sinus	Isolated	ATD

Abbreviations: Rt, right; Lt, left; NA, not available; ATD, anti-tubercular drugs.

## Case presentation

A 14-year-old boy, from a low socio economic background presented with a four-month history of pain, and a discharging sinus in the right upper scapular region that had been present for two months. The pain had been gradual, dull and aching. The patient had been treated for these complaints without relief and had developed a scapular swelling which broke down and discharged serosanguinous fluid. He had an antecedent history of trauma and an associated history of fever, weight loss, loss of appetite, night sweats, malaise and fatigue. He had no history of previous pulmonary or extrapulmonary tuberculosis and there was no family history of tuberculosis.

**Figure 1:**
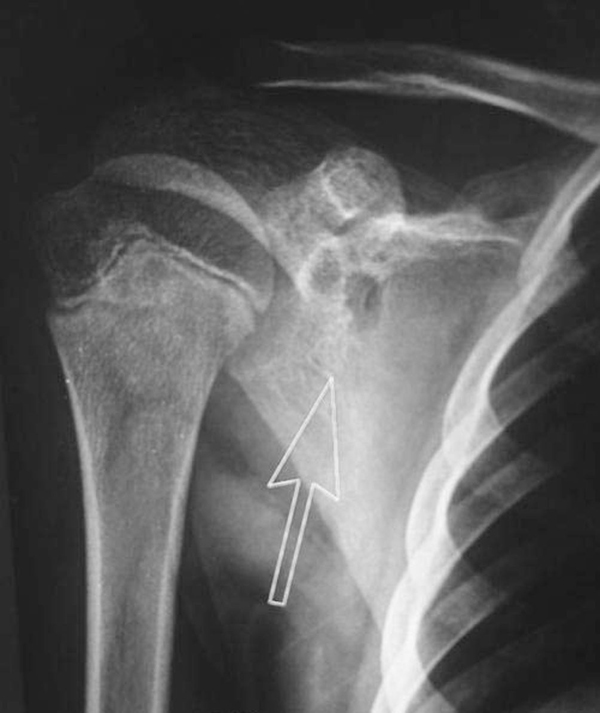
**Anteroposterior (AP) radiograph of the shoulder showing two well defined lytic destructive lesions involving the glenoid margin suggestive of cystic tuberculosis**.

**Figure 2:**
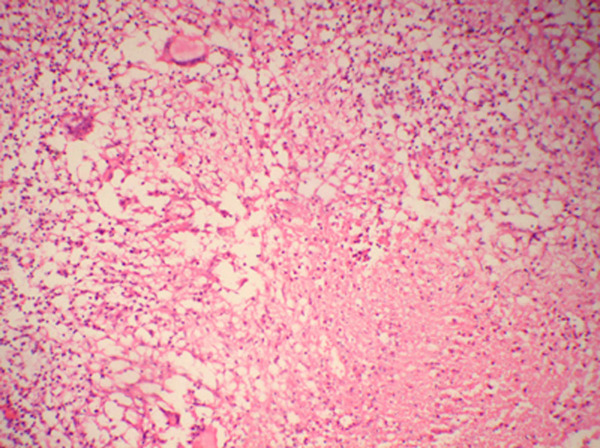
**Microphotograph showing epithelioid cell granulomas with necrosis**. H&E ×40.

On local examination, we observed a sinus measuring less than 1 cm in size overlying the right upper scapular region. It was slightly tender, adherent to the bone and surrounding soft tissue, with associated granulation tissue and serosanguinous discharge and the surrounding skin was indurated and unhealthy. There was no significant regional lymphadenopathy, he had a full range of motion of the shoulder joint and there was no tenderness over the spine and paraspinal muscles in the thoracic region. Laboratory examination showed only a minimally increased white blood cell count (10950/mm^3^) with a predominance of lymphocytes (48%), elevated erythrocyte sedimentation rate (ESR) of 65 mm (Westergren method) after one hour and a positive C-reactive protein (CRP) test. A Mantoux tuberculin skin test (purified protein derivative, five tuberculin units) was positive with 15 mm of induration observed 48 hours after administration. Anteroposterior radiographs of the right shoulder showed two rounded oval lytic areas with low density seen in the body of the scapula involving the glenoid margin (Figure 1) and there was a minimal increase in density surrounding the lesion. A plain chest radiograph was normal and a closed core biopsy of the sinus tract revealed epithelioid cell granulomas with central necrosis, typical Langhans giant cells and a positive stain for acid fast bacilli by Ziehl-Neelsen stain (Figure 2). On microbiologic examination positive culture on Lowenstein-Jenson medium for AFB was present. Anti-tuberculosis chemotherapy began immediately. The patient received four months of anti-tubercular chemotherapy, consisting of four drugs: isoniazid (INH), pyrazinamide, ethambutol and rifampicin. He was given INH, rifampicin and ethambutol for four months and INH and rifampicin for 10 months. Radiographs at 10 months showed complete resolution of the bony lesion. The sinus healed without any complications after four months of anti-tubercular treatment. The patient's appetite improved, he gained weight and his growth indices significantly improved at the end of the anti-tubercular treatment. At two-year follow-up he was asymptomatic.

## Discussion

Osteoarticular tuberculosis accounts for 3% of all cases of tuberculosis and isolated tuberculosis of the scapula is rare. In past reports most cases were associated with other forms of tubercular osteomyelitis and only six were isolated to the scapula [5]-[9,12]. We report tuberculosis of the scapula in a 14-year-old male patient. Previously, Greenhow and Weintrub [10] also reported tubercular involvement of the scapula in a pediatric patient. Clinically, patients with osteoarticular tuberculosis present with localized symptoms of swelling and pain as was present in our case. Radiograph of the shoulder showed a well defined lytic destructive lesion of the scapula indicative of cystic tuberculosis. Cystic tuberculosis is a rare form of tuberculosis seen mostly in children and young adults, usually in the appendicular skeleton; occasionally involving flat bones as seen in the present case. Cystic tubercular involvement of the scapula has only once been reported, in the literature [5] and there seems to be a changing pattern of cyst-like lesions in osseous tuberculosis. Multicystic and multifocal lesions were more common 50 years ago, but it seems that solitary lesions are now predominant and this may be related to immunological factors. Vohra et al. [8] detected nine solitary cystic lesions in six adults and three children. In the present case we found two cystic lesions near the glenoid margin of the scapula. Bone lesions were usually solitary because of sensitization of the patient to the tubercle bacillus; however, if host immunity is poor and the immune response has been altered, the lesions may multiply. Trauma probably draws the attention to a mild focus or it may activate a latent tubercular focus. Sinus formation and abscess are common in tuberculous osteitis as seen in our case. The diagnosis of tuberculosis was based on the staining of smears for acid-fast bacilli and culturing for mycobacteria. AFB smear results lack sensitivity and are not specific for tuberculosis [13] and while mycobacterial culture and identification is specific for diagnosis, it takes two to three weeks. Histologic diagnosis in conjunction with microbiologic and molecular testing should be considered appropriate for the diagnosis.

## Conclusion

Although rare, tuberculosis should be suspected in patients presenting with a chronic sinus in the scapular region, particularly in the developing world. As uncommon presentations and sites of osteoarticular disease can be a source of delay and error in management, an open biopsy may be necessary in doubtful cases.

## Abbreviation

TB: tuberculosis.

## Consent

Written informed consent was obtained from the patient's parent for publication of this case report and any accompanying images. A copy of the written consent is available for review by the Editor-in-Chief of this journal.

## Competing interests

The authors declare that they have no competing interests.

## Authors' contributions

All of the authors were involved in examination of the patient as well as in writing and reviewing the manuscript.
